# Countertransference feelings and personality disorders: a psychometric evaluation of a brief version of the Feeling Word Checklist (FWC-BV)

**DOI:** 10.1186/s12888-020-02556-6

**Published:** 2020-03-30

**Authors:** R. Breivik, T. Wilberg, J. Evensen, J. I. Røssberg, H. S. J. Dahl, G. Pedersen

**Affiliations:** 1 Division of Mental Health and Addiction, Oslo University Hospital, P.O. Box 4959, Nydalen, 0424 Oslo, Norway; 2 University of Oslo, Institute of Clinical Medicine, P.O. Box 1171, Blindern, 0318 Oslo, Norway; 3 Division of Mental Health and Addiction, Vestfold Hospital Trust, P.O. Box 2168, 3103 Tønsberg, Norway; 4 Division of Mental Health and Addiction, Oslo University Hospital, Nydalen Outpatient Clinic, P.O. Box 4925, 0424 Oslo, Norway; 5 Division of Mental Health and Addiction, Department of Personality Psychiatry, Oslo University Hospital, Oslo, Norway; 6 NORMENT, KG Jebsen Center for Psychosis Research, Institute of Clinical Medicine, University of Oslo, Oslo, Norway

**Keywords:** Countertransference, Feeling Word Checklist, Factor analysis, Personality disorder, Psychometrics

## Abstract

**Background:**

The Feeling Word Checklist (FWC) is a self-report questionnaire designed to assess therapists’ countertransference (CT) feelings. The primary aim of the study was to evaluate the psychometric properties of a brief, 12-item version of the Feeling Word Checklist (FWC-BV). The second aim was to validate the factor structure by examining the associations between the FWC-BV factors, patients’ personality pathology and therapeutic alliance (TA).

**Methods:**

Therapists at 13 different outpatient units within the Norwegian Network of Personality Disorders participated, and the study includes therapies for a large sample of patients (*N* = 2425) with personality pathology. Over a period of 2.5 years, therapists completed the FWC-BV for each patient in therapy every 6 months. Statistical methods included exploratory (EFA) and confirmatory (CFA) factor analysis. Internal consistency was estimated using Mc Donald’s coefficient Omega (ω_t_). The Structured Clinical Interview for DSM-IV – Axis II (SCID II) and Mini International Neuropsychiatric Interview (MINI) were used as diagnostic instruments, and patient-rated TA was assessed using the Working Alliance Inventory (WAI-SR).

**Results:**

Factor analyses revealed three clinically meaningful factors: *Inadequate*, *Idealised* and *Confident*. These factors had acceptable psychometric properties. Most notably, a number of borderline PD criteria correlated positively with the factors *Inadequate* and *Idealised*, and negatively with the factor *Confident*. All the factors correlated significantly with at least one of the WAI-SR subscales.

**Conclusions:**

The FWC-BV measures three clinically meaningful aspects of therapists’ CT feelings. This brief version of the FWC seems satisfactory for use in further research and in clinical contexts.

## Background

Sigmund Freud first introduced the term *countertransference* (*CT*) to refer to an analyst’s transference to the patient [1]. That is, therapists unconsciously displace feelings from their past onto analytic situations. Ideally, this is not supposed to happen; analysts are supposed to stay calm and objective, allowing no personal material to interfere with therapy. In this narrow Freudian view, CT is essentially an obstacle to be overcome, arising from therapists’ own unresolved conflicts. Freud’s solution was for therapists to undergo more analyses to become aware of and gain control over such potentially disturbing feelings. In the 1950s, the concept of CT broadened. It was now seen as a road to knowledge about the patients’ problems, as patients communicate something important about their inner world with the feelings they induce in their therapist. In this broader view, CT is understood as all the feelings evoked in the therapist, both conscious and unconscious [2, 3]. Later, the concept has been further modified with what is referred to as relational psychoanalysis and is here seen more as a co-created phenomenon [4–6]. According to Gabbard [7, 8], CT has expanded to encapsulate both the narrow and the broad view. However, there is still a controversy within the psychoanalytic community as to what countertransference is and what role it plays during treatment. In this study, CT is defined as the therapist’s emotional response to the patient, that is, the feelings evoked when treating a patient.

Although the concept of CT originally derives from psychoanalytic theory, several current psychotherapeutic approaches regard the therapist’s emotional reactions as an important aspect of the therapeutic process [9–11]. However, historically, empirical work on CT has been sparse compared to the enormous amount of theoretical literature written about the phenomenon. A main challenge in the development of systematic research has been the lack of a common definition of *CT*. Additionally, CT is conceptualized as a partially unconscious process, and therefore difficult to assess. What can be measured are its manifestations. In the present study, we used a questionnaire (FWC-BV) designed to capture the therapist’s self-reported feelings. That is, the feelings and experiences the therapists become aware of, acknowledge, remember and are willing to report after sessions [12]. With self-reports, we only get access to the conscious manifestations of CT. Thus, we might only capture a small part of the CT phenomenon with this method. From a psychoanalytic perspective, it is theorized that it is the feelings that we are not aware of that often drive us to act. Patients with personality disorders (PD) may particularly evoke strong emotions in the therapist and in severe cases CT can contribute to derailing treatment. However, clinical experience suggests that by focusing on CT, we can see that the same patterns in the therapeutic dyad are repeated over and over again. The therapist may then gradually become more aware of CT [12]. Despite the obvious weakness that self-reports only capture the conscious aspects of CT, recent studies have concluded that self-reports can measure CT phenomena in clinically sophisticated and psychometrically sound ways [9, 13, 14]. Additionally, questionnaires can provide a method of capturing information about the treatment process, which may help clinicians make diagnostic and therapeutic use of their own response to the patient [9, 13, 14].

Two approaches have been used to measure CT empirically. One is to have therapists fill out self-report questionnaires [13, 15]. The other is to have an external observer evaluate recorded material from sessions [16–18]. An advantage of self-reports is their quantitative nature; they can be distributed to many therapists, and one can subsequently aggregate large amounts of data enabling identification of common patterns of feelings [9]. The Feeling Word Checklist (FWC) [15, 19, 20], in various versions, is one of the most used questionnaires for research on CT feelings [21]. A general disadvantage of self-reports is, nevertheless, the so-called defensive bias. Therapists may find it difficult to report negative feelings. Additionally, they may not be aware of such feelings and thus unable to report them. In this paper, only the conscious affective responses are measured, and these responses are seen as a part of the total CT construct. Other researchers have focused on different CT manifestations. So far, researchers have operationalized the manifestations along behavioural, cognitive and affective levels [10].

### The FWC

The instrument provides a list of different feeling words and therapists register if or to which extent these have been experienced in relation to a patient in a therapy session. The first version of the FWC was developed by Whyte et al. [15] and comprised 30 feeling words. Later, different versions have included different item numbers ranging from 24 to 58 feeling words. The different versions aimed, partly, to include feeling words which experienced therapists found lacking in the original list [12] and partly, to enhance the stability of the underlying factors in the FWC. Different factors underlying the items of the FWC have been identified, and between three and seven factors are described. Variations in the number of factors may be explained by different FWC versions and dissimilar scale formats, such as Likert scales or dichotomous yes/no versions [20].

Different statistical methods have been used to evaluate FWC. Most studies have used principal component analyses, some are based on factor analyses [22]. Furthermore, the studied samples have been heterogeneous, involving therapists of diverse professions, varying sample sizes, and different patient populations. The first studies using FWC were performed in inpatient departments. More recent studies have examined the factor structure when FWC is applied in individual therapy [12, 22–24]. There is still no consensus about which FWC version best captures the CT phenomenon. Generally, all studies have found at least one factor reflecting positive feelings and at least one reflecting negative feelings [22]. One goal in the CT research is to find many of the same feeling factors, as this may support that important aspects of CT have been captured. So far, many of the factors in the FWC overlap.

The present study investigates a brief FWC version (FWC-BV) featuring only 12 feeling words. A brief instrument is easier to implement in clinical contexts, thus facilitating the psychotherapy process and outcome research. An instrument such as the FWC-BV can also help therapists become more aware of their feelings. Several studies have demonstrated that therapists’ feelings are related to patient outcomes [11, 25]. For therapists to become aware of this phenomenon, it must be given attention. There is a need for research on the psychometric properties of instruments used in CT research to make future studies more robust [26]. A brief questionnaire, such as the FWC-BV, will obviously not capture all important CT feelings, but it can provide insight into meaningful aspects or patterns of feelings during therapy processes.

### CT and personality disorder

There is extensive clinical and theoretical literature on CT and patients with PD, and a considerable relational strain is often reported by therapists. Particularly, many clinical articles are about borderline PD. Kernberg [27] described that these patients tend to elicit powerful CT reactions in therapists because of their intense, primitive, and regressive transferences. Furthermore, it has been argued, that specific CT feelings are the most reliable guide to diagnose borderline PD. These include the feeling of being idealised or devaluated as a therapist. There are, however, few empirical studies on PD and CT [9, 14, 28–30]. Two studies have explored therapists’ feelings in relation to Diagnostic and Statistical Manual of Mental Disorders Version 4 (DSM-IV) diagnoses at the PD cluster level and found that that patients with cluster A (i.e., paranoid, schizoid and schizotypal PD) and B disorders (i.e., antisocial, borderline, histrionic and narcissistic PD) elicited more negative CT feelings than did patients with cluster C disorders (i.e., avoidant, dependent and obsessive compulsive PD) [9, 28]. Some studies [9, 14] have found that specific PD categories elicit different CT feelings in therapists. For example, in the study by Colli et al. [14], borderline PD was associated with emotional response feelings such as *Helpless*/*Inadequate*, *Overwhelmed*/*Disorganised* and *Special*/*Overinvolved*, while avoidant PD was associated with *Parental*/*Protective* and *Special*/*Overinvolved* responses. This study indicated that therapists’ feelings can nearly be applied diagnostically. A few studies have looked at PD dimensionally in terms of the number of fulfilled PD criteria and CT feelings. Dahl et al. [12], for example, found a strong negative relationship between the total number of fulfilled PD criteria and confident CT.

### CT and therapeutic alliance (TA)

The term *therapeutic alliance* (*TA*) refers to the working relationship between the therapist and patient. To date, only a few studies have investigated the relationship between TA and CT [12, 18, 23, 26, 31]. Both the TA measurement instruments and results of these studies vary widely. Existing studies also differ in terms of whether TA is patient-rated, therapist-rated or based on both perspectives. To summarise, two studies reported a negative correlation between negative aspects of CT feelings and TA [18, 26], while others found both negative correlations between negative CT feelings and TA and positive correlations between positive CT feelings and TA [12, 23, 31].

### Aims of the present study

Many former studies of CT are based on small, heterogeneous patient populations, and very few have investigated therapies with poorly functioning PD patients with PD. This study consists of a large sample of patients with significant PD pathology. In this way, it may be possible to examine the feelings that therapists can experience while working with a varied sample of PD patients.

The primary aim of the current study is to explore the factor structure and psychometric properties of the FWC-BV, used in a clinical sample of patients with PD or PD traits. Our secondary aim is to validate these factors by examining their relationship with patients’ personality pathology and TA.

More specifically, we wanted to answer the following research questions:

1. How many clinically meaningful factors do the items in the FWC-BV represent?
2. What is the relationship between therapists’ CT feelings, assessed by FWC-BV, and patients’ personality pathology?
3. What is the relationship between therapists’ CT feelings, assessed by FWC-BV, and patient-rated TA?

## Method

### Participants

#### Treatment units

In this multi-site, naturalistic and explorative study, data was collected from 13 outpatient units within the Norwegian Network of Personality Disorders [32] in the period 2010 to 2016. All units were outpatient services on a specialist mental health service level, providing treatment for a broad range of patients with significant personality problems and personality disorder (PD). The different units combined psychoeducational, group and individual psychotherapy formats and treatment approaches were mainly psychodynamic, but combinations also included body awareness, art and cognitive therapies. Specific PD approaches implemented within some units in the Network during the investigation period, included mentalization-based therapy, dialectical behaviour therapy and schema-focused therapy. Treatment was usually time-limited, and most units had an upper time limit between 2 and 3 years.

The different treatment units collected patient data and the therapists’ self-report questionnaires (FWC-BV), which were registered in an anonymous central database, administrated by the Department for Personality Psychiatry, Oslo University Hospital.

#### Therapists

In this study, all therapist data were anonymous and the number of therapists participating was unknown. However, some general information was available: The multidisciplinary therapist teams usually included psychiatrists, psychologists, psychiatric nurses and social workers. Most of the therapists are formally trained (for 3 to 5 years) in group analytic psychotherapy [32, 33]. The Network regularly provides updated courses and conferences on PD assessment procedures and therapeutic principles [32, 34]. Based on current information from 10 of the 13 units, the mean number of therapists at each unit was 10, approximately 75% female and 25% male. Mean age was 45 years. Therapists had a mean age of 17 years of clinical experience, and 73% of the therapists had education in group psychotherapy. Group supervision of the therapists is traditionally an important element in treatment programs, and CT is part of the clinical discussions. The therapists filled out the FWC-BV at 6-month intervals for each patient they had in treatment (i.e., from 6 months up to 2.5 years), with a final assessment at end of the patient’s treatment. A total of 4849 FWC-BV were completed during the study period.

#### Patients

The sample consisted of 2425 adult patients. They were referred to treatment within the specialist mental health service on a regular basis, from a primary health service level. The mean age was 33 years (standard deviation [SD] = 10 years), and 76% of the patients were female. According to the guidelines given in DSM-IV [35], 71% of participants had one or more PD diagnosis and 94% had at least one symptom disorder, wherein 68% had mood disorders and 57% had anxiety disorders (see Table 1 for prevalence of PDs). The severity of PD is illustrated through different outcome measures: the Global Assessment of Functioning (GAF; APA, 1994) and Work and Adjustment Scale (WSAS) [36] measure patient psychosocial functioning; the Global Severity Index (GSI) measures symptom distress and is the mean score of the Revised Symptom Checklist-90 (SCL-90-R) [37]; and the Index of Interpersonal Problems (IIP) measures interpersonal problems and is the mean score of the Circumplex of Interpersonal Problems (CIP) [38]. The CIP is a revised version of the Inventory of Interpersonal Problems – Circumplex (IIP-C) [39].

**Table 1:** Prevalence of PD and range of PD criteria

***Prevalence of PDs***		***Median number of PD criteria (range)***
Paranoid	158 (7%)	1 (0–7)
Schizoid	9 (0%)	0 (0–5)
Schizotypal	1 (0%)	0 (0–7)
Antisocial	14 (1%)	0 (0–5)^a^
Borderline	489 (23%)	2 (0–9)
Histrionic	1 (0%)	0 (0–5)
Narcissistic	10 (1%)	0 (0–8)
Avoidant	721 (33%)	2 (0–7)
Dependent	82 (5%)	1 (0–7)
Obsessive-compulsive	113 (5%)	0 (0–7)
PD NOS	345 (16%)	8 (0–22)
No PD	626 (29%)	0 (0–16)
PD diagnosis deferred	258 (11%)	–

*PD NOS* personality disorder not otherwise specified

^a^Adult antisocial criteria

In the current study, the mean GAF score was 49.77 (SD = 6.06), and according to APA [35] within the “Sever” range. Mean WSAS score was 22.60 (SD = 8.56), and according to Mataix-Cols and colleagues [40] and Pedersen and colleagues [41] in the “Moderate” range. In addition, the GSI was 1.54 (SD = 0.66) and IIP was 1.65 (SD = 0.52). With respect to GSI this score is in the “Moderate” to “Sever” range [37, 42, 43], and a score of 1.65 on CIP is associated with severe interpersonal distress [42, 44]. Thus, all these measures reflect a poorly functioning patient group with a high level of symptom and interpersonal distress. TA was measured using the revised short form of the Working Alliance Inventory (WAI-SR). The patients filled out the WAI-SR at the same intervals as did the therapists when filling out the FWC-BV (i.e., every 6 months from 6 months up to 2.5 years in their treatment period, with a final assessment at the end of the treatment).

### Assessment

#### The FWC

The FWC is a self-report measure in which therapists rate their emotional responses toward a patient in a five-point response format (0–4), ranging from ‘No such feeling’ (0) to ‘Very much’ (4). The present study uses a brief version (FWC-BV) of the Feeling Word Checklist 58 (FWC-58) that includes 12 items. In the FWC-BV, the prompt, ‘During recent conversations with the patient I have felt...’ is followed by the 12 feeling words: *Disliked*, *Important*, *Threatened*, *Exalted*, *Bored*, *Confident*, *Inadequate*, *Admired*, *On Guard*, *Calm*, *Invaded* and *Overview*. Each of the words is rated from 0 to 4 by therapists, based on how strongly they experience each feeling.

The FWC-BV is new and was constructed for this study with the aim of creating a more applicable and less time-consuming questionnaire, reflecting some positive and some negative feelings. The aim in its creation is to determine whether these positive and negative feelings are important cues to describe therapy processes and outcomes in future studies. The items were selected from the FWC-58. The selection was data-driven based on former factor analysis of the FWC-58 from a large heterogeneous material comprising data from different in- and outpatient clinics [12, 20]. We selected the 12 items with the strongest loadings in the factor structure of the FWC-58; six items with positive feelings and six items with negative feelings. The 12 items were discussed and evaluated by an experienced researcher and clinician for clinical relevance in the present study.

#### Diagnostics

All patients were diagnosed according to DSM-IV [35] using the Mini International Neuropsychiatric Interview (MINI) [45] for symptom disorders and the Structured Clinical Interview for DSM-IV – Axis II (SCID-II) for PD. [46] Diagnostic reliability was not investigated. However, diagnostic assessments were performed in each unit by clinical staff who had received systematic training in diagnostic interviews and principles of the Longitudinal, Expert, All-Data (LEAD) procedure [47, 48]. This means that diagnoses were based on all available information, including referral letters, self-reported history, complaints, overall clinical impression and the results of the two diagnostic interviews (i.e., the MINI and SCID-II). In DSM-IV, the classification of PD is polythetic—that is, the criteria within each disorder are neither necessary nor sufficient. The number of fulfilled PD criteria can thus be seen as a reflection of the dimensional strength or closeness to prototypic PD constructs.

#### TA

The patients filled in the WAI-SR [49, 50] every 6 months during treatment and at discharge from treatment. The WAI-SR is a 12-item questionnaire representing 3 different aspects of the patient’s relationship to the therapist; bond, task and goal. Patients are asked to judge each question on a Likert scale from ‘Never’ (1) to ‘Always’ (7). The patients filled out two versions of the WAI-SR: one with reference to their group therapist (WAI-G) and one with reference to their individual therapist (WAI-I).

### Statistics

#### Unbalanced data

The data of the current study are based on ordinary routine assessments, but it is important to note that these routines sometimes are disturbed for one reason or another. Therapists may sometimes fail to fill in FWC-BV according to the time schedule, and administrative routines may be hampered so that patients do not receive six-month questionnaires. As such, the dataset in the current study is unbalanced. See Table 2 for an assessment of the FWC-BV and WAI-SR. To check for possible patient-therapist bias, patients who were evaluated on FWC-BV were compared to those patients not evaluated on FWC-BV at the time of 12 months of therapy. No significant differences were found on GAF, WSAS, GSI, CIP, or the number of fulfilled PD criteria. Thus, we found no indication of systematic bias threatening to the validity of the study results.

**Table 2:** FWC-BV assessments at different times

***Number of FWC-BV assessments completed by therapists***	
6 months	806
1 year	869
1.5 years	606
2 years	412
2.5 years	266
End of treatment	1890

#### Factor analysis

We decided to analyse the FWC-BV after 12 months in therapy, assuming that therapy is well underway by that point. There is usually also some delay from the initial assessment period to inclusion in the treatment programme, although most patients have individual clinical contact with the unit during this waiting time. As such, there is good reason to assume that the treatment process is stabilised 1 year after the initial assessment.

The total sample of 2425 patients was first randomly divided into 2 separate sub-samples. This was done to facilitate the exploratory (EFA) and confirmatory factor analysis (CFA). The first sub-sample (*n* = 1219) was used to conduct explorative factor analyses and the second (*n* = 1206) to cross-validate the suggested factor structure in a confirmatory factor analysis. After 1 year of therapy, the number of available completed FWC-BV questionnaires was 869. With respect to the initial factor analysis, sub-sample 1 (for EFA) comprised 439 FWC-BV questionnaires and sub-sample 2 (for CFA) comprised 430 FWC-BV questionnaires. All other analyses are based on the total sample of 2425 patients.

Using IBM SPSS Statistics for Windows, Version 25 (2017), randomisation of the total sample was done with the Select function (approximate 50%). Relationships between variables were estimated by Pearson Product-Moment Correlation, and scale reliability was estimated by McDonald’s Omega (ω_t_) [51, 52]. EFA and CFA were conducted using Mplus 7.11 (Muthén & Muthén, Los Angeles, CA, USA) [53], with estimations based on the maximum likelihood (ML) and maximum likelihood mean (MLM) adjusted functions, respectively. The mean-adjusted chi-square test statistic, also referred to as the Satorra–Bentler chi-square [54] is robust to non-normality.

To evaluate the CFA models, goodness of fit was estimated by root mean square error of approximation (RMSEA) [55], the non-normed fit index (NFI) [56]—also called the Tucker Lewis Index (TLI) [57]—the comparative fit index (CFI) [58] and the standardised root mean square residual (SRMR) [59].

An RMSEA of 0.05 or below indicates a good model fit, values between 0.05 and 0.08 indicate a reasonable fit, values between 0.08 and 0.10 indicate a mediocre fit and values above 0.10 indicate an unacceptable fit [60]. However, a cut-off value close to 0.06 [59] or a stringent upper limit of 0.07 [61] seem to be the general consensus of what is considered acceptable. The TLI and CFI both measure model fit in comparison to the independence model. Both are derived from the chi-square statistic and are supposed to lie between 0 and 1. Values greater than 0.90 for these measures are normally required for good fit of a model, although Hu and Bentler [59] have suggested TLI ≥ 0.95 as the threshold. The SRMR is the mean absolute value of the covariance residuals, and it ranges from 0 to 1. Well-fitting models should obtain values less than 0.05 [62, 63], but values up to 0.08 are acceptable [59].

## Results

### Factor analyses

#### Factor structure of the FWC-BV

The EFA of sub-sample 1 (*n* = 439 at 1 year) indicated four factors with eigenvalues above 1.0, accounting for 67% of the observed variance. The fifth factor had an eigenvalue of 0.716, accounting for 6% of the remaining observed variance. The CFA of sub-sample 2 (*n* = 430 at 1 year), based on the suggested four-factor model from the EFA, had a chi-square model fit of 99.533 (degrees of freedom [df]: 48; *p* = 0.0000) and an RMSEA (90% confidence interval [CI]), CFI, TLI and SRMR of 0.050 (0.036–0.064), 0.948, 0.929 and 0.054, respectively, indicating perfect fit. A subsequent CFA based on the sub-samples 1 and 2 combined (*n* = 869 at 1 year) also revealed good model fit. However, factor three comprised only two items (*Bored* and *Inadequate*), and *Bored* had a high degree of residual variance (86%). Moreover, one item of factor two (*Threatened*) was distinguished by a considerable lack of variance (mean: 0.05; SD: 0.28; Skewness: 6.24; Kurtosis: 45.46)—that is, hardly a strongly expressed feeling. Based on these findings, *Bored* and *Threatened* were omitted from the item pool, and *Inadequate* was moved to factor two. The solution was then a three-factor solution based on 10 items/feelings, labelled *Idealised*, *Inadequate* and *Confident*. See Table 3 for the final operationalisation of the three-factor model and estimates of scale reliabilities. As shown in Table 3, the scale reliabilities are in the acceptable range (i.e., mainly between .70 and .80) except for the factor *Inadequate*.

**Table 3:** Three-factor model of FWC-BV assessed 1 year after initial assessment and estimates of scale reliability at each assessment point

	***Factors and loadings based on one-year evaluations***		
***Items / feeling words***	*Idealised*	*Inadequate*	*Confident*
*Important*	0.575		
*Exalted*	0.867		
*Admired*	0.851		
*Disliked*		0.573	
*Inadequate*		0.615	
*On Guard*		0.576	
*Invaded*		0.568	
*Confident*			0.722
*Calm*			0.876
*Overview*			0.581
***Evaluations***	***Mc Donald’s coefficient Omega (ω***_***t***_***) (95% CI)***		
6 months	0.82 (0.80–0.84)	0.64 (0.58–0.70)	0.75 (0.71–0.79)
1 year	0.80 (0.77–0.82)	0.67 (0.63–0.72)	0.79 (0.75–0.82)
1.5 years	0.81 (0.78–0.84)	0.67 (0.61–0.73)	0.80 (0.76–0.83)
2 years	0.79 (0.76–0.83)	0.65 (0.59–0.71)	0.79 (0.74–0.84)
2.5 years	0.79 (0.74–0.83)	0.72 (0.63–0.81)	0.79 (0.74–0.84)
End of treatment	0.81 (0.80–0.83)	0.72 (0.69–0.75)	0.79 (0.77–0.81)

*CI* confidence interval

From the CFA of the new three-factor model, it was reasonable to conduct two modifications. The first was to accept a negative cross-loading from the item *Important* to the factor *Inadequate*, and the second was to accept a negative residual covariance between the item *Inadequate* with the item *Overview* of the factor *Confident*. In Table 4, model fit indices are shown for all assessment periods from 6 months to end of treatment. From this, all fit indices indicate excellent to good fit to the model, except from FWC evaluations at 2.5 years. The main reason for this indication of misfit was a cross-loading of the item *Invaded* with the factor *Idealised*. By accepting this cross-loading in a modified specification, the model fit was found to be acceptable (RMSEA = 0.074; CFI = 0.937; TLI = 0.902). Based on these considerations, we concluded that this three-factor structure based on 10 items revealed the best model.

**Table 4:** Goodness-of-fit statistics from CFA based on the three-factor model of FWC-BV questionnaires at different time points

***Evaluations***	***X***^***2***^***(df)***	***RMSEA (90% CI)***	***CFI***	***TLI***	***SRMR***
6 months	83.37 (30)	0.047 (0.035–0.060)	0.969	0.954	0.043
1 year	91.92 (30)	0.049 (0.038–0.061)	0.971	0.957	0.037
1.5 years	114.34 (00)	0.069 (0.055–0.082)	0.944	0.917	0.050
2 years	76.56 (30)	0.062 (0.045–0.079)	0.956	0.934	0.053
2.5 years	84.16 (30)	0.083 (0.062–0.104)	0.918	0.877	0.060
End of treatment	214.86 (30)	0.058 (0.050–0.065)	0.965	0.948	0.044

Chi-square statistics: *p* values < .0001

*CFA* confirmatory factor analysis, *CFI* comparative fit index, *CI* confidence interval, *RMSEA* root mean square error of approximation, *SRMR* standardised root mean square residual, *TLI* Tucker-Lewis Index

### Validity

Table 5 shows the mean levels of the three FWC-BV scales across gender and selected PDs. The mean values after 1 year of therapy was highest for *Confident* (mean = 2.74; SD = 0.74), followed by *Idealised* (mean = 1.12; SD = 0.78) and *Inadequate* (mean = 0.47; SD = 0.50).

**Table 5:** Descriptive statistics of FWC-BV subscales

	***Idealised*** ***Mean (SD)***	***Inadequate*** ***Mean (SD)***	***Confident*** ***Mean (SD)***
***Total sample***			
Total sample	1.12 (0.78)	0.47 (0.50)	2.74 (0.74)
Males	1.11 (0.80)	0.50 (0.50)	2.73 (0.72)
Females	1.12 (0.78)	0.47 (0.50)	2.74 (0.75)
***Diagnostic sub-groups***^a^			
No PD (*n* = 195)	1.06 (0.74)	0.40 (0.44)	2.84 (0.72)
Paranoid PD (*n* = 62)	1.15 (0.82)	0.60 (0.60)	2.72 (0.63)
Borderline PD (*n* = 232)	1.27 (0.87)	0.62 (0.58)	2.59 (0.76)
Avoidant PD (*n* = 289)	1.06 (0.76)	0.44 (0.48)	2.77 (0.73)
Dependent PD (*n* = 40)	1.16 (0.71)	0.45 (0.35)	2.80 (0,77)
Obsessive-Compulsive PD (*n* = 50)	0.97 (0.69)	0.55 (0.61)	2.65 (0.79)
PD NOS	1.13 (0.77)	0.47 (0.46)	2.71 (0.68)

FWC assessed at 1 year

*PD NOS* PD not otherwise specified

^a^No correction for comorbidity among PDs; only PDs represented by more than ten cases were listed

To further validate the CT factors, we explored the relationship between the factors, the number of PD criteria, and TA. As shown in Table 6, *Confident* correlated negatively with the total number of PD criteria. *Inadequate* had a positive correlation with total number of PD criteria, and the borderline, narcissistic and paranoid PD criteria. *Idealised* showed a positive correlation with borderline and histrionic PD criteria. The avoidant PD criteria showed a weak, but not significant, positive correlation with *Confident*.

**Table 6:** Correlations between the FWC-BV and other clinical measures

	***Idealised***	***Inadequate***	***Confident***
***Number of PD criteria***			
Paranoid	0.020	0.137^**^	−0.046
Schizoid	−0.066	0.007	− 0.091^**^
Schizotypal	0.006	0.066	− 0.104^**^
Antisocial (adult criteria)	0.033	0.074^*^	0.006
Borderline	0.101^**^	0.245^**^	−0.168^*^
Histrionic	0.116^**^	0.057	−0.030
Narcissistic	0.035	0.125^**^	− 0.075^**^
Avoidant	− 0.054	− 0.054	0.017
Dependent	0.020	0.044	−0.044
Obsessive-Compulsive	0.007	0.064	−0.011
Total number of PD criteria^a^	0.046	0.171^**^	− 0.117^**^
***Working Alliance Inventory***			
Evaluation of group therapists (WAI-G)			
Goals	0.019	−0.196^**^	0.124^**^
Tasks	0.028	−0.211^**^	0.153^**^
Bond	−0.023	−0.185^**^	0.113^**^
Evaluation of individual therapists (WAI-I)			
Goals	0.086^*^	−0.260^**^	0.160^**^
Tasks	0.119^**^	−0.226^**^	0.190^**^
Bond	0.071	−0.264^**^	0.193^**^

FWC and WAI assessed at 1 year

*Correlation is significant at the 0.05 level (2-tailed)

**Correlation is significant at the 0.01 level (2-tailed)

^a^Antisocial PD criteria for child and adulthood omitted

Patient-rated TA showed a positive correlation with *Confident* and a negative correlation with *Inadequate*. *Idealised* had a positive correlation with evaluation of the individual therapists (WAI-I) but not with evaluation of the group therapists (WAI-G).

## Discussion

The main objective of the current study was to investigate the factor structure and psychometric properties of the FWC-BV. We found 10 items that constituted three CT factors, namely, *Idealised*, *Inadequate* and *Confident*. The factors were psychometrically acceptable and clinically recognisable and can be seen as aspects of CT feelings that can be evoked when treating PD patients.

There are conceptual similarities between the present factors and several of those found in other studies. Holmquist et al. [19] were the first to examine the underlying factor structure of FWC applied in individual psychotherapy. They used an FWC with 48 feeling words (FWC-48) and found four factors to be evoked: *Positive*, *Negative*, *Distant* and *Dejected*. The factor *Positive* seems to show similarities with the factor *Confident* in our study. *Negative* and *Dejected* seem to show similarities with *Inadequate*. Dahl et al. [12] used a version with 58 feeling words (FWC-58) and found four factors; two of these (*Confident* and *Inadequate*) conceptually overlap with our study. The same holds for the factors *Confident* and *Inadequate* in the study by Ulberg et al. [23] (FWC-24), and the factor *Inadequate* in that of Lindquist et al. [22] (FWC-24). Further, in studies where the setting is inpatient care, Røssberg et al. [20] found seven factors, two of which (*Inadequate* and *Confident*) conceptually overlap with ours (FWC-58). However, we cannot say that the factors mentioned above are directly comparable, as different studies have used different FWC questionnaires [23] and included different patient populations. *Idealised* is a factor not reported in previous studies on FWC, but similarities between some of the previous factors reported in other studies, such as *Important* in the study by Røssberg et al. [20], can be identified. Nevertheless, few studies on FWC seem to have captured this aspect of CT. For an overview of previous FWC studies, see Lindquist et al. [22].

It is noteworthy that the therapists overall score a low intensity of their CT feelings. The mean scores range from 0.47 (*Inadequate*) to 1.12 (*Idealised*) to 2.74 (*Confident*). The scores are in a similar range to those seen in many other FWC studies — that is, quite low scores are consistently reported [12, 19, 22, 23]. However, as the patients in the present study are mainly poorly functioning PD patients, one might have expected stronger feelings to be reported. It is also somewhat surprising that *Confident* is the feeling assigned the highest score. This could be due to the therapists overall being highly experienced and the fact that regular group supervision is part of the therapists’ work, in which CT is a focus. This is in line with the report of Ulberg et al. [23], in which *Confident* was positively associated with more experience as well as with an increased level of supervision — that is, they found lower levels of *Inadequate* feelings with more supervision.

Alternatively, the result could also be due to defensive processes in therapists. Some may find it difficult to report negative feelings. The therapist also might not be aware of more negative feelings. As mentioned earlier, CT is partly an unconscious phenomenon, at least initially, and sometimes we only become aware of it through enactments. This is also why treatments of severe personality disorders are in greater danger of failing. However, one could expect that therapists in this study are more aware of their CT feelings as they often have supervision on it. Another explanation might be that the questionnaires were only filled in every 6 months. Some therapists have reported that ‘overall’ they feel relatively confident in meeting with the patient when they look at the relationship over a long period of time. More frequent measurements could likely capture more varied and possibly more intense CT feelings.

The three factors, *Idealised*, *Inadequate* and *Confident*, are consistent with aspects of feeling responses identified in clinical literature on psychotherapy with PD patients [64, 65] and in the existing empirical literature [9, 14]. Interestingly, we specifically found that in therapies with patients meeting many borderline PD criteria — therapists felt both more idealised and more inadequate. We also found that the total number of PD criteria correlated positively with the *Inadequate* factor. This is in line with the report of Dahl et al. [12].

In this study, feelings evoked in therapies with patients with avoidant PD were not clearly identified in the FWC-BV. Avoidant PD and borderline PD constitute the two largest patient groups in this material. Avoidant PD criteria showed a weak, but not significant, positive correlation with a *Confident* response. Previous empirical studies have reported that *Confident* is the most significant response from the group in question [21]. However, this result might also reflect that Avoidant PD patients induce greater variation in what therapist feel when treating these patients. From a clinical perspective, there is reason to believe that therapists may experience more negative feelings than previous studies have reported, especially toward more poorly functioning avoidant PD patients.

Another explanation might be that the CT response had more to do with the severity of personality pathology in terms of the number of PD criteria across specific disorders. Some researchers have questioned the validity of PDs as categorical constructs, as high PD co-occurrence exist. Sharp et al. [66] evaluated a bi-factor model for PD pathology in which a general factor and several specific factors of personality pathology account for the covariance among PD criteria. In particular, they studied the borderline PD criteria and found that they loaded only on the general factor which may suggest that BPD criteria represent core features of PD severity. Thus the nine BPD criteria may represent higher levels of disturbed behaviour. Whether the CT responses toward PD patients can be better explained by general factors is an interesting question, but beyond the scope of this paper.

Correlational analyses with patient-rated TA also revealed several meaningful and significant associations. Patient-rated TA, measured using the WAI-SR, showed a positive correlation with *Confident* and a negative correlation with *Inadequate*. This is in line with the study by Dahl et al. [12], although they rated patients’ TA with a different instrument, called the Help and Understanding Scale (HUS). The correlations between patient-rated TA and CT are of particular interest because of the non-overlapping perspectives. *Idealised* correlated positively with patients’ evaluation of their individual therapist (WAI-I) but not with their group therapist (WAI-G). From a clinical experience, it could be speculated that the individual therapist is idealised more than the group therapist. In treatment programs involving group therapy, patients must share a therapist’s attention with up to seven other group members, making way for more complicated feelings such as envy, feelings of exclusion and feelings of being alone among others; as such, the therapist’s lack of omnipotence is more striking than it is in individual sessions. As far as is known, no other empirical studies have found this association.

### Strengths and limitations

A considerable strength of this study is that the sample was large enough to be divided in two, for EFA and subsequent CFA, and that each of the sub-samples were large enough to yield stability in the estimates. Another strength is that the data comprise a large and representative sample of patients with PD and PD traits, wherein PD not otherwise specified (NOS), borderline PD and avoidant PD are the most prevalent diagnoses. The patient sample is also well described, representing a functionally impaired and highly symptomatic patient group —a group known to evoke powerful CT reactions in therapists. No previous studies on PD and CT have investigated CT using such a large sample of poorly functioning patients. This study can thus contribute to highlight feelings that are typically described in clinical literature but only to a small extent, empirically investigated. However, the poorly functioning patients in this study may also restrict the generalisation of the results to more well-functioning, clinical samples.

One of the main limitations is the lack of information of the number of therapists participating in the study. Hence, we do not know how many patients each therapist may have treated or if the same therapist has scored the FWC for the same patient at all assessment times (i.e. every 6 months for the same patient). Thus, there is some interdependence in the data. A strength is that data from 13 different treatment units in Norway were collected, and there is reason to believe that the number of therapists is relatively high due to the number of units assessed. We also know that the staff working in the various units are relatively stable, that is, they have typically worked for several years within the same units.

### Implications

This study has important clinical and research applications. As far as known, no brief version of CT questionnaires exists in the field. A brief version is easy to introduce in clinical contexts and also has several research advantages. It is easier to make repeated measurements of therapists feelings, e.g. after every session with a patient. Thus, this instrument can give a better understanding of how CT contributes in the process and outcome of therapy.

PD patients often trigger problematic countertransference reactions in therapists - and the risk of acting out in the course of treatment is higher [27]. Hence, focus on CT is particularly relevant with these patients. In this study, comprising units specialized in the treatment of PDs a focus on CT is already part of the daily work, and systematic supervision is recommended. One might assume that the level of CT feelings would be more intense or problematic in other sections of the public health system, and hence, the importance of becoming aware of CT manifestations is even higher in other areas of the health services. A brief questionnaire like FWC-BV is a short and easy instrument that can help clinicians across theoretical orientations to become more aware of their feelings during the course of treating patients.

There is a growing literature that underscores the importance of building a good working relationship with the patients [67–69]. Research findings demonstrate that alliance rupture repair is associated with positive psychotherapy outcome [68]. A brief instrument to measure CT feelings together with other process variables such as alliance can possibly give a better understanding of rupture-repair sequences in the therapeutic relationship. How do negative feelings affect the therapist and treatment outcome? If negative feelings are not taken seriously or are acted out they may affect the commitment to the patient, and the willingness to recognize and repair alliance ruptures. These are clinically interesting questions that should be addressed in future studies.

## Conclusion

In this study, we found that the FWC-BV comprised three factors based on ten of the twelve items, labelled *Inadequate*, *Idealised* and *Confident*. The subscales had satisfactory internal consistency and were meaningfully related to patients’ personality pathology and TA. Thus, this shorter list of feeling words seems to identify common experiences evoked in meetings with patients with PD and may prove valuable for further research, as well as for use in clinical and educational contexts.

## Data Availability

Due to restrictions imposed by the Regional Medical Ethics Committee regarding patient confidentiality, data are available upon request. Requests for data may be sent to the hospital’s Privacy and Data Protection Officer at: personvern@ous-hf.no

## Abbreviations

CFA: Confirmatory factor analysis
CFI: Comparative fit index
CIP: Circumplex of interpersonal problems
CT: Countertransference
df: Degree of freedom
DSM-IV: Diagnostic and statistical manual of mental disorders version 4
EFA: Exploratory factor analysis
FWC-BV: Feeling Word Checklist brief version
FWC: Feeling Word Checklist
GAF: Global Assessment of Functioning
GSI: Global Severity Index
HUS: Help and Understanding Scale
IIP: Index of Interpersonal Problems
LEAD: Longitudinal, Expert, All-Data
MIIC: Mean Inter-Item Correlation
M.I.N.I.: Mini International Neuropsychiatric Interview
ML: Maximum likelihood
MLM: Maximum Likelihood Mean Adjusted
NFI: Non-normed fit index
ω_t_: Mc Donald’s coefficient Omega
PD: Personality disorder
PD NOS: Personality disorder not otherwise specified
RMSEA: Root mean square error of approximation
SCID-II: Structured Clinical Interview for DSM-IV – Axis II Personality Disorders
SCL-90-R: Revised Symptom Checklist
SD: Standard deviation
SRMR: Standardised root mean square residual
TLI: Tucker Lewis Index
WAI-G: Working Alliance Inventory – Group Therapist
TA: Therapeutic alliance
WAI-SR: Short version of Working Alliance Inventory
WAI-I: Working Alliance Inventory – Individual Therapist
WSAS: Work and Adjustment Scale
